# Rasmussen's Encephalitis: A Report of a Tunisian Pediatric Case and Literature Review

**DOI:** 10.1155/2020/6810237

**Published:** 2020-06-24

**Authors:** Hedia Klaa, Thouraya Ben Younes, Hanene Benrhouma, Sonia Nagi, Aida Rouissi, Ichraf Kraoua, Ilhem Ben Youssef-Turki

**Affiliations:** ^1^Department of Child and Adolescent Neurology, Research Laboratory LR18SP04, National Institute Mongi Ben Hmida of Neurology, Tunis, Tunisia; ^2^University of Tunis El Manar, Faculty of Medicine of Tunis, Tunis 1007, Tunisia; ^3^Department of Neuroradiology, National Institute Mongi Ben Hmida of Neurology, Tunis, Tunisia

## Abstract

Rasmussen's encephalitis (RE) is a rare progressive inflammatory disease of the central nervous system. It is characterized by unilateral hemispheric atrophy, pharmacoresistant focal seizures, and progressive neurological deficit. The exact etiopathogenesis still remains unknown. Brain imaging plays an important role in diagnosis and follow-up. Fluctuation of lesions in brain imaging was reported in few cases. Herein, we report an additional pediatric case of Rasmussen encephalitis with fluctuating changes in brain MRI.

## 1. Introduction

Rasmussen's encephalitis (RE) was first described in the late 1950s. It is a rare neurological disease of childhood characterized by unilateral hemispheric atrophy, pharmacoresistant focal seizures, and progressive neurological deficits. The exact etiopathogenesis still remains unknown. Brain imaging plays a pivotal role in diagnosis and control of disease progression. Few cases with atypical MRI features of RE represented by improvement and reoccurrence of signal abnormalities were reported.

We report on the clinical, electrophysiological, and imaging data of an additional pediatric case of RE with atypical MRI features.

## 2. Case Study

**Figure 1:**
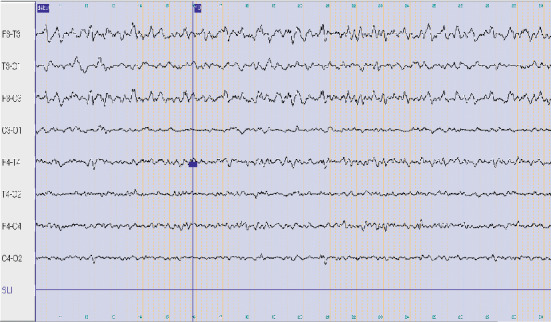
EEG showing asymmetric background activity with the left frontotemporal intercritical discharges.

**Figure 2:**
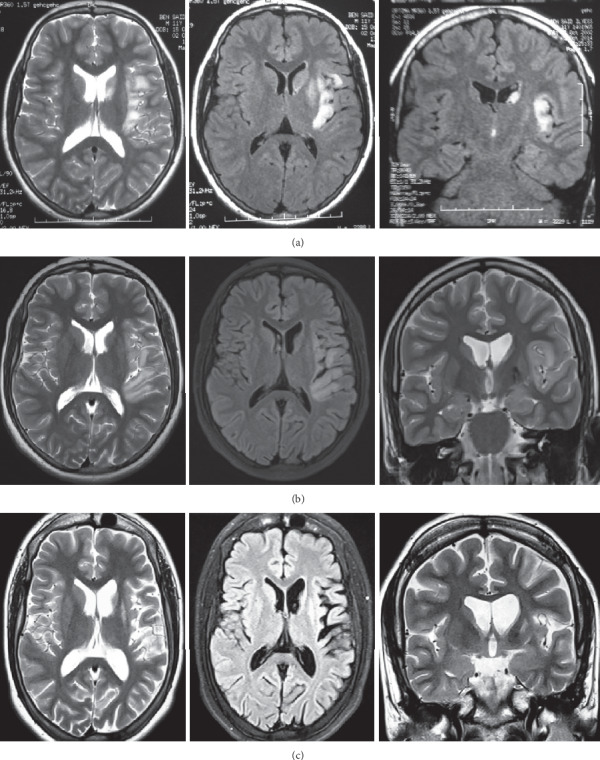
Serial MRIs in T2 and FLAIR sequences showing hyperintensity in the left frontoinsular region, lenticular, and caudate nuclei (a). Seven months after the first MRI, persistence of hyperintensity, left fronto-insular cortical and homolateral striatum atrophy, and dilatation of the ipsilateral ventricular system (b). After 3 years and 9 months from the first MRI, we noticed an increase of the left hemispheric atrophy (c).

An 11-year-old boy was born to nonconsanguineous parents. He had a family history of febrile seizures in his sister, epilepsy in 2 cousins, and ulcerative colitis in his mother. He had no significant antenatal and perinatal history. Psychomotor development was normal. He was treated for adrenal insufficiency and dysthyroïdism. On August 2014, he was referred to our department, at the age of 11, with focal clonic right-sided seizures, which were preceded by gastroenteritis 1 month ago. Neurological examination showed right hemidystonia, myoclonia, right pyramidal syndrome, and right hemihypoesthesia. Interictal electroencephalogram (EEG) showed left frontotemporal discharge persisting during sleep (Figure 1). Brain magnetic resonance imaging (MRI) showed cortical and subcortical hyperintensity on T2-weighted (T2) and fluid-attenuated inversion recovery (FLAIR) images in the left frontoinsular region, homolateral lenticular, and caudate nuclei (Figure 2(a)). Spine MRI was normal. Initially, the diagnosis of acute disseminated encephalomyelitis (ADEM) was suspected. Routine blood and cerebrospinal fluid investigations were normal. Infectious serologies (HSV, CMV, EBV, HIV, VZV, HVC, HVB, syphilis, and Lyme) and immunological assessment (ANA, anti-DNA, ANCA, APL, ACL, anti-B2GP1, and anti-ENA) were negative. The patient received a pulse of steroids (1 g/day) during 5 days and, then, relay per os at the dose of 1 mg/kg/day for 10 days. Valproic acid and clobazam were prescribed with clinical improvement and partial seizure control. A second brain MRI performed after 3 weeks was normal. On April 2015, he presented with intractable focal right seizures, progressive impairment of language abilities, and behavioral disorders with irritability, eating disorders (polyphagia), and worsening school performance with working memory problems. Neurological examination showed right hemiparesis and dystonia of the right upper limb. Given the fluctuating subacute course, seizures, behavioral disturbances, and progressive cognitive impairment, autoimmune encephalitis was suspected. Serum and CSF screening for an anti-N-methyl-D-aspartate (NMDA) receptor, anti-leucine-rich glioma-inactivated protein1 (LGi1), anti-contactin-associated protein-2 (Caspr2), anti-2-amino-3-(3-hydroxy-5-methylisoxazol-4-yl), propanoic acid (anti-AMPA), anti-GABAa and anti-GABAb, anti-glycine, anti-amphiphysin, anti-Hu, anti-Yo, anti-Ri, anti-CV2, anti-Ma1, and anti-GAD were negative. The brain MRI showed cortical hyperintensity on T2 and FLAIR images in left frontoinsular, left frontoinsular cortical atrophy with homolateral striatum atrophy, and dilatation of the ipsilateral ventricular system (Figure 2(b)). Given the clinical course and MRI finding, the diagnosis of RE was performed. Numerous regimens of antiepileptic drugs were prescribed (valproic acid 2 g/day, carbamazepine 1400 mg/day, levetiracetam 2500 mg/day, clonazepam 4 mg/day, and piracetam 1600 mg/day). A monthly steroid pulse at a dose of 1 g/day for 3 days was administered during 12 months. Azathioprine was prescribed on January 2017 at the dose of 100 mg/day. Partial control of seizures was obtained. Nevertheless, he presented with several status epilepticus concomitant to infectious episodes. The motor function improved mildly. A control of the brain MRI was performed on September 2017 and showed an increase of the left hemispheric atrophy (Figure 2(c)).

## 3. Discussion

We report the case of an 11-year-old boy presenting with focal seizures. The diagnosis of Rasmussen's encephalitis (RE) was made due to the clinical and radiological findings. Our patient illustrates a rare case of RE with fluctuating signal abnormalities on brain MRI.

RE is a progressive chronic inflammatory disease of the central nervous system. It was first reported by Theodore Rasmussen in 1958 [1]. The disorder is rare and affects mostly children. The median age of onset is 6 years. Our patient had a late-onset RE. Both sexes are equally affected [2, 3]. It is characterized by focal intractable seizures, progressive neurological deficit, and cognitive decline, with unihemispheric brain atrophy, found in our patient [2]. The etiopathogenesis of RE is unknown. Suggested etiologies include viral infections, an autoimmune phenomenon involving circulating antibodies against glutamate receptors, and cytotoxic T cells [3, 4]. Diagnosis of RE is based on characteristic clinical, radiological, and pathological features. Diagnostic criteria were established by Bien et al. in 2005 [5]. Three stages have been proposed. The prodromal stage is manifested with mild signs (low seizure frequency and mild hemiparesis). The acute stage is characterized by frequent focal seizures, progressive hemiparesis, and cognitive deterioration. The residual stage is characterized by stabilization of neurological deficits and continuation of seizures, but less frequent than in the acute stage [6]. In some cases, less common presentations such as unilateral movement disorders, including hemiathetosis and hemidystonia, have been reported [7]. Our patient had right hemidystonia.

Brain MRI is an important tool for diagnostic assessment and follow-up in RE [2, 5]. The majority of patients show, at an earlier stage, unilateral enlargement of the ventricular system which is accentuated in the insular and periinsular regions. A T2/FLAIR hyperintense signal is often present in the cortical or subcortical regions. Afterwards, unihemispheric atrophy sets in and predominates typically in the perisylvian region, as in our case. Most of the tissue loss happens during the first 12 months after the onset of symptoms in the majority of patients. Atrophy of the ipsilateral head of the caudate nucleus is a typical feature. Gadolinium enhancement is very rare in RE [5]. Serial MRIs demonstrate progression of signal change and atrophy. Improvement and one reoccurrence of signal abnormalities represent an atypical MRI feature of RE. Reappearance of high signal intensity was associated with clinical seizure aggravation, as observed in our case. This fluctuation is indicative of the inflammatory process [8]. In the literature, 9 cases of RE showed regression followed by reappearance of lesions on serial MRIs [4, 8–10]. Only 3 of them had high signal intensity lesions at initial examination, similar to our observation [8]. In line with the fluctuating nature of the MRI changes, we evoked, initially, the diagnosis of ADEM.

In early disease stages, electroencephalograms (EEG) may contribute to the diagnosis of RE. Various abnormalities are seen in patients with RE. Some unihemispheric findings such as impairment of background activity with persistent polymorphic delta waves and sleep spindles, focal slow activity, subclinical ictal discharges, and multifocal ictal discharges are strongly suggestive of RE [2, 3]. The EEG of our patient showed intercritical left frontotemporal discharge persisting during sleep.

Brain biopsy can also help the diagnosis, but it is not required in all RE cases. The characteristic histopathological features are microglial and lymphocytic nodules, neuronal loss, neuronophagia, and perivascular cuffing, confined to one cerebral hemisphere with frontoinsular predilection [2, 5].

RE can be treated by antiepileptic drugs, immunosuppressive and immunomodulator regimens, and surgery. The aim of these treatments is to reduce seizure severity and improve the motor and cognitive performance [2, 5]. Frequently, seizures are resistant to antiepileptic drugs [11]. Patients receiving immunotherapy had a beneficial effect on seizure frequency and delayed deterioration [12]. Steroids, prescribed in our case, are the most effective treatment and the most widely used. Pulses of high-dose methylprednisolone have been reported to be effective to stop disease progression [11].

Intravenous immunoglobulin (IVIG) was used in some patients having RE with good results. The recommended dose is 2 g/kg monthly. The association of steroids and IVIG may be indicated when the two treatments alone are ineffective [13].

Plasmapheresis has good effects on seizures and neurological functions. It contributes to assessing the mental and residual motor function before surgery. The frequency was three to six single volume exchanges on consecutive or alternate days every 2 to 8 weeks [13].

Other medical treatments, such as using tacrolimus, rituximab, cyclophosphamide, azathioprine, and interferon, have been reported [11]. Our patient was treated by azathioprine.

Surgery (anatomic hemispherectomy, functional hemispherectomy, perisylvian hemispherotomy, trans-sylvian hemispherotomy, and central/vertical hemispherotomy) seems to be the only cure for the seizures and to improve cognitive outcome. However, inevitable sequelae (hemianopia, hemiparesis, and aphasia in the dominant hemisphere) should be considered [2]. Rehabilitation approach should be considered. It may improve the quality of life of RE patients.

## 4. Conclusions

Rasmussen's encephalitis is a progressive inflammatory disease of one cerebral hemisphere characterized by frequent focal seizures, hemiparesis, and mental deterioration. Brain imaging findings, associated with electroencephalogram and clinical data, may indicate early diagnosis and could be an indicative of prognosis. Knowledge of atypical radiological aspects is necessary in order to establish the right diagnosis. Rapid diagnosis and management can modify the progression of disease.

## Data Availability

No data were used to support this study.
